# Chronic kidney disease is associated with increased risk of sudden sensorineural hearing loss and Ménière’s disease: a nationwide cohort study

**DOI:** 10.1038/s41598-021-99792-x

**Published:** 2021-10-12

**Authors:** Jong-Yeup Kim, Suehyun Lee, Jaehun Cha, Gilmyeong Son, Dong-Kyu Kim

**Affiliations:** 1 Department of Otorhinolaryngology-Head and Neck Surgery, College of Medicine, Konyang University, Daejeon, Republic of Korea; 2 Department of Biomedical Informatics, College of Medicine, Konyang University, Daejeon, Republic of Korea; 3 Department of Otorhinolaryngology-Head and Neck Surgery, Chuncheon Sacred Heart Hospital, Hallym University College of Medicine, 77, Sakju-ro, Chuncheon-si, Gangwon-do 24253 Republic of Korea; 4 Institute of New Frontier Research, Division of Big Data and Artificial Intelligence, Hallym University College of Medicine, Chuncheon, Republic of Korea

**Keywords:** Diseases, Health care, Risk factors, Urology

## Abstract

Several studies have demonstrated the harmful effects of chronic kidney disease (CKD) on the audiovestibular system. Through a time-to-event analysis, we aimed to compare the association of CKD with sudden sensorineural hearing loss (SSNHL), and Ménière’s disease against a control population without CKD. We used a total of 1,025,340 patients from the Korean National Health Insurance Service database from 2002 to 2013. The CKD group (n = 2572) included patients diagnosed with CKD more than three times between January 2003 and December 2005. The non-CKD group (n = 5144) consisted of two patients without CKD for every patient with CKD. Each patient was monitored until December 2013. We calculated the incidence, survival rate, and hazards ratio (HR) of SSNHL and Ménière’s disease. In the CKD group, the incidence of SSNHL and Ménière’s disease was 1.39 and 3.64 per 1000 person-years, respectively. Patients with CKD showed an adjusted HR of 2.15 and 1.45 for SSNHL and Ménière’s disease, respectively. Middle-aged patients with CKD were associated with a higher incidence of developing SSNHL and Ménière’s disease than those without CKD. Female patients with CKD had a higher risk of developing SSNHL; however, there was no significant difference in the risk of Ménière’s disease in patients with CKD according to sex. Our findings suggest that CKD is associated with an increased incidence of SSNHL and Ménière’s disease. Therefore, audiovestibular surveillance should be considered in patients with CKD.

## Introduction

Chronic kidney disease (CKD) is defined as abnormalities of kidney structure or function that are present for > 3 months and have an impact on health^1^. The global prevalence of CKD is estimated to range from 11 to 13%, and it may be more common than diabetes mellitus (DM) with an estimated prevalence of approximately 8%^2^. According to the 2012 Kidney Disease Improving Global Outcomes clinical practice guidelines, CKD is divided into five classes based on the value of the glomerular filtration rate (GFR). Increasing evidence has shown that CKD is an independent risk factor for cardiovascular disease, and there is an inverse relationship between GFR and risk of cardiovascular disease^2–5^. Additionally, prior studies have revealed that decreased renal function might be a predictor of hospitalization, cognitive dysfunction, and poor quality of life^3,6–8^. Patients with CKD could suffer from several otorhinolaryngological problems, including sensorineural hearing loss, epistaxis, candidiasis, halitosis, xerostomia, dysgeusia, and lip, and thyroid cancers^9^.

Among otorhinolaryngological complications, dysfunction of the audiovestibular system is one of the most common problems^10,11^. The etiology of these complications in patients with CKD may be due to several structural and functional similarities between the kidney and the inner ear^12^. Moreover, disturbances in water and electrolyte homeostasis can affect the endolymphatic fluid, which may induce endolymphatic hydrops^13^. Finally, some drugs such as loop diuretics and aminoglycosides used in the treatment of patients with CKD are well-known for their ototoxicity^14^. To date, only a few studies using population-based data have demonstrated a relationship between CKD and dysfunction of the audiovestibular system^15,16^. One study has shown that patients with CKD are at a higher risk of developing sudden sensorineural hearing loss (SSNHL) than the general population^15^. Another study has demonstrated that patients with CKD are at an increased risk of developing vestibular dysfunction in comparison to healthy populations^16^. Thus, CKD may be considered a risk factor for SSNHL or Ménière’s disease. However, studies on the relationship between CKD and SSNHL or Ménière’s disease are still lacking.

Therefore, to determine a significant link between CKD and SSNHL or Ménière’s disease, we investigated the prospective hazard of SSNHL and Ménière’s disease in patients with CKD using a nationwide representative sample from the National Sample Cohort (NSC) 2002–2013 data provided by the Korea National Health Insurance Service (KNHIS) in South Korea.

## Results

**Figure 1:**
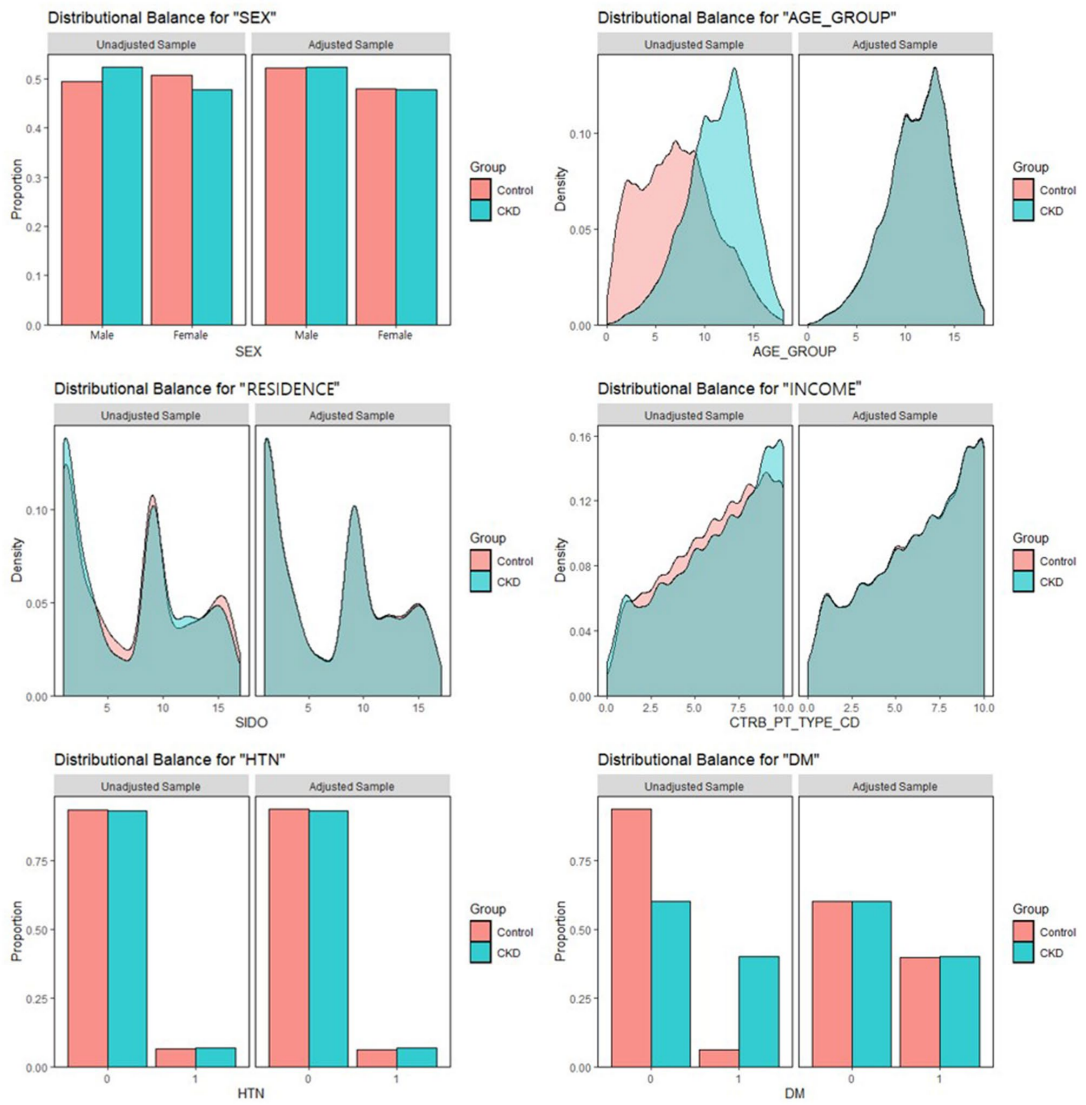
Balance plot for 6 variables before and after matching.

We selected 2572 patients with CKD and 5144 comparison participants (non-CKD) using propensity score matching. The details of the study population and group characteristics are presented in Table 1. We observed no significant differences between the CKD and comparison groups regarding sex, age, residential area, household income, hypertension (HTN), or diabetes mellitus (DM). This indicates that group matching was performed appropriately. Additionally, to indicate good matching, we presented the balance plot before and after matching (Fig. 1). This showed a good matching balance along with our sensitivity analysis.

**Table 1:** Characteristics of the study participants.

Variables	Comparison (n = 5144)	Chronic kidney disease (n = 2572)	*P* value
**Sex**			0.917
Male	2682 (52.1%)	1345 (52.3%)	
Female	2462 (47.9%)	1227 (47.7%)	
**Ages (years)**			0.970
< 45	1408 (27.4%)	709 (27.6%)	
45–64	2409 (46.8%)	1197 (46.5%)	
> 64	1327 (25.8%)	666 (25.9%)	
**Residence**			0.983
Seoul (metropolitan)	1247 (24.2%)	625 (24.3%)	
2nd area (other metropolitan)	1300 (25.3%)	654 (25.4%)	
3rd area	2597 (50.5%)	1293 (50.3%)	
**Household income**			0.987
Low (0–30%)	1044 (20.3%)	522 (20.3%)	
Middle (30–70%)	1885 (36.6%)	938 (36.5%)	
High (70–100%)	2215 (43.1%)	1112 (43.2%)	
**Hypertension**			0.261
No	4820 (93.7%)	2392 (93.0%)	
Yes	324 (6.3%)	180 (7.0%)	
**Diabetes mellitus**			0.967
No	3094 (60.1%)	1545 (60.1%)	
Yes	2050 (39.9%)	1027 (39.9%)	

SSNHL, Sudden sensorineural hearing loss; Seoul, a largest metropolitan; 2nd area, other metropolitan cities; 3rd area, other areas.

In this study, we examined a total of 78,915.7 person-years and 78,453.3 person-years of SSNHL and Ménière’s disease, respectively, which included 56,559.9 person-years and 56,172.7 person-years in the comparison group, and 22,355.8 person-years and 22,280.6 person-years in the CKD group, respectively. Our results showed that the incidence of SSNHL and Ménière’s disease per 1000 person-years was 1.39 and 3.64, respectively, for the CKD group, compared to 0.60 and 3.38, respectively, for the comparison group.

**Figure 2:**
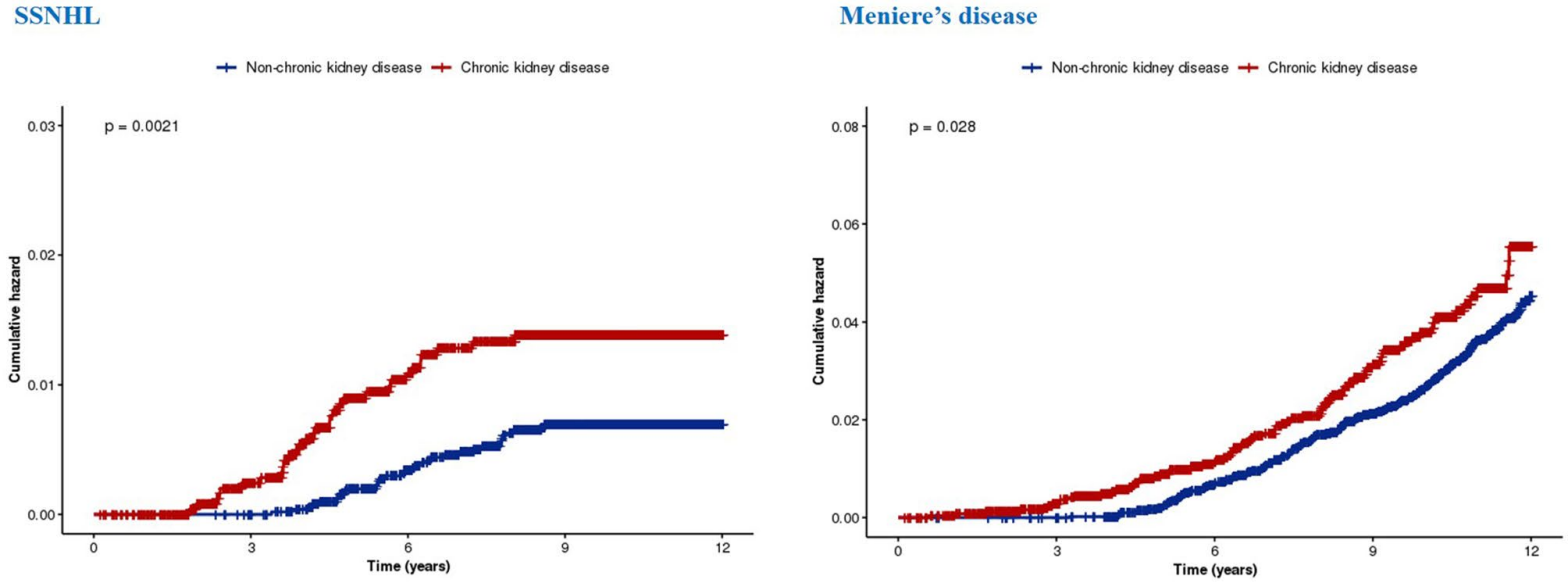
Risk of development of SSNHL and Ménière’s disease among individuals with and without chronic kidney disease. SSNHL, sudden sensorineural hearing loss.

We analyzed the hazards ratios (HR) for the development of SSNHL and Ménière’s disease during the 8-year follow-up period using univariate and multivariate Cox regression models; these findings are presented in Tables 2 and 3. After adjusting for sociodemographic factors (sex, age, residential area, household income, hypertension [HTN], and DM), we observed that patients with CKD had a significant association with the prospective development of SSNHL (adjusted HR = 2.15, 95% confidence interval [CI] [1.32–3.51]) and Ménière’s disease (adjusted HR = 1.45, 95% CI [1.11–1.89]). Additionally, we observed that HTN and DM were significantly associated with the prospective development of SSNHL (HTN, adjusted HR = 2.38, 95% CI [1.17–4.85]; DM, adjusted HR = 3.18, 95% CI [1.87–5.42]), whereas the adjusted HR of Ménière’s disease was significantly increased among the females and older age groups (women, adjusted HR = 1.48, 95% CI [1.16–1.88]; patients aged > 64 years, adjusted HR = 2.94, 95% CI [2.00–4.31]). Moreover, the reverse Kaplan–Meier survival curves and the results of log-rank tests are presented in Fig. 2, which depicts the cumulative incidences of SSNHL and Ménière’s disease in the patient and comparison groups.

**Table 2:** Incidence per 1000 person-years and hazards ratio (95% confidence interval) of sudden sensorineural hearing loss.

Variables	N	Case	Person-years	Incidence	Unadjusted HR (95% CI)	*P* value	Adjusted HR (95% CI)	*P* value
**Group**								
Comparison group	5144	34	56,559.9	0.60	1 (ref)		1 (ref)	
CKD group	2572	31	22,355.8	1.39	2.11 (1.30–3.43)**	0.003	2.15 (1.32–3.51)**	0.002
**Sex**								
Male	4027	37	40,408.2	0.92	1 (ref)		1 (ref)	
Female	3689	28	38,507.5	0.73	0.81 (0.49–1.32)	0.388	0.79 (0.48–1.30)	0.352
**Ages (years)**								
< 45	2117	16	22,783.0	0.70	1 (ref)		1 (ref)	
45–64	3606	34	37,789.3	0.90	1.30 (0.71–2.35)	0.394	0.99 (0.53–1.83)	0.965
> 64	1993	15	18,343.4	0.82	1.18 (0.58–2.38)	0.653	0.89 (0.43–1.84)	0.748
**Residence**								
Seoul	1872	16	19,202.5	0.83	1 (ref)		1 (ref)	
2nd area	1954	15	20,064.3	0.75	0.90 (0.44–1.82)	0.768	0.93 (0.46–1.89)	0.849
3rd area	3890	34	39,648.9	0.86	1.03 (0.57–1.87)	0.915	0.99 (0.54–1.80)	0.972
**Household income**								
Low (0–30%)	1566	11	15,696.4	0.70	1 (ref)		1 (ref)	
Middle (30–70%)	2823	26	29,105.5	0.89	1.29 (0.64–2.60)	0.483	1.26 (0.62–2.55)	0.525
High (70–100%)	3327	28	34,113.8	0.82	1.18 (0.59–2.38)	0.634	1.09 (0.54–2.20)	0.812
**Hypertension**								
No	7212	56	73,764.4	0.76	1 (ref)		1 (ref)	
Yes	504	9	5151.3	1.75	2.33 (1.16–4.72)*	0.018	2.38 (1.17–4.85)*	0.016
**Diabetes mellitus**								
No	4639	22	48,118.6	0.46	1 (ref)		1 (ref)	
Yes	3077	43	30,797.1	1.40	3.09 (1.85–5.17)***	< 0.001	3.18 (1.87–5.42)***	< 0.001

CKD, Chronic kidney disease; SSNHL, Sudden sensorineural hearing loss; HR, Hazard ratio; CI, Confidence interval. *P < 0.05, **P < 0.010, and ***P < 0.001.

**Table 3:** Incidence per 1000 person-years and hazards ratio (95% confidence interval) of Meniere’s disease.

Variables	N	Case	Person-years	Incidence	Unadjusted HR (95% CI)	*P* value	Adjusted HR (95% CI)	*P* value
**Group**								
Comparison group	5144	190	56,172.7	3.38	1 (ref)		1 (ref)	
CKD group	2572	81	22,280.6	3.64	1.34 (1.03–1.75)*	0.028	1.45 (1.11–1.89)**	0.006
**Sex**								
Male	4027	111	40,304.9	2.75	1 (ref)		1 (ref)	
Female	3689	160	38,148.4	4.19	1.48 (1.16–1.88)**	0.002	1.48 (1.16–1.88)**	0.002
**Age (years)**								
< 45	2117	41	22,794.3	1.80	1 (ref)		1 (ref)	
45–64	3606	143	37,632.3	3.80	2.13 (1.51–3.02)***	< 0.001	2.06 (1.45–2.94)***	< 0.001
> 64	1993	87	18,026.7	4.83	2.98 (2.05–4.32)***	< 0.001	2.94 (2.00–4.31)***	< 0.001
**Residence**								
Seoul	1872	53	19,147.5	2.77	1 (ref)		1 (ref)	
2nd area	1954	72	19,928.0	3.61	1.31 (0.92–1.87)	0.134	1.39 (0.98–1.99)	0.068
3rd area	3890	146	39,377.9	3.71	1.35 (0.99–1.85)	0.062	1.34 (0.98–1.84)	0.068
**Household income**								
Low (0–30%)	1566	52	15,658.4	3.32	1 (ref)		1 (ref)	
Middle (30–70%)	2823	104	28,970.4	3.59	1.06 (0.76–1.48)	0.731	1.14 (0.81–1.58)	0.457
High (70–100%)	3327	115	33,824.5	3.40	1.01 (0.73–1.40)	0.962	1.01 (0.73–1.41)	0.937
**Hypertension**								
No	7212	254	73,305.2	3.46	1 (ref)		1 (ref)	
Yes	504	17	5148.1	3.30	0.94 (0.57–1.53)	0.797	0.88 (0.54–1.45)	0.625
**Diabetes mellitus**								
No	4639	141	47,895.4	2.94	1 (ref)		1 (ref)	
Yes	3077	130	30,557.9	4.25	1.46 (1.15–1.86)**	0.002	1.25 (0.98–1.60)	0.074

CKD, Chronic kidney disease; SSNHL, Sudden sensorineural hearing loss; HR, Hazard ratio; CI, Confidence interval. *P < 0.05, **P < 0.010, and ***P < 0.001.

Next, we performed subgroup analyses to examine the association between CKD and the development of SSNHL or Ménière’s disease according to age and sex. After adjusting for other variables, we observed that the prospective development of SSNHL and Ménière’s disease was only significantly related to the middle-aged CKD group (45–64 years; SSNHL, adjusted HR: 2.36, 95% CI [1.29–4.62]; Ménière’s disease, adjusted HR: 1.50, 95% CI [1.04–2.14]; Table 4). Additionally, we observed that female patients with CKD were more strongly associated with the development of SSNHL than male patients (Table 5). However, there was no significant relationship between Ménière’s disease and CKD according to sex (Table 4).

**Table 4:** Hazard ratios of sudden sensorineural hearing loss or Meniere’s disease by age between patients with and without chronic kidney disease.

Variables	N	Case	Person–years	Incidence	Unadjusted HR (95% CI)	*P* value	Adjusted HR (95% CI)	*P* value
**SSNHL**								
**Age (years) < 45**								
Comparison group	1408	8	6948.9	0.51	1 (ref)		1 (ref)	
CKD group	709	8	15,834.1	1.15	2.08 (0.78–5.54)	0.143	2.05 (0.77–5.48)	0.152
**Age (years) 45**–**64**								
Comparison group	2409	17	27,034.5	0.63	1 (ref)		1 (ref)	
CKD group	1197	17	10,754.8	1.58	2.27 (1.16–4.45)*	0.017	2.36 (1.20–4.62)*	0.012
**Age (years) > 64**								
Comparison group	1327	9	13,691.3	0.66	1 (ref)		1 (ref)	
CKD group	666	6	4652.1	1.29	1.82 (0.64–5.11)	0.259	1.92 (0.68–5.42)	0.220
**Meniere’s Diseases**								
**Age (years) < 45**								
Comparison group	1408	28	15,850.1	1.77	1 (ref)		1 (ref)	
CKD group	709	13	6944.2	1.87	1.21 (0.62–2.36)	0.572	1.22 (0.63–2.38)	0.561
**Age (years) 45**–**64**								
Comparison group	2409	98	26,914.0	3.61	1 (ref)		1 (ref)	
CKD group	1197	45	10,718.3	4.20	1.48 (1.03–2.12)*	0.033	1.50 (1.04–2.14)*	0.029
**Age (years) > 64**								
Comparison group	1327	64	13,408.6	4.77	1 (ref)		1 (ref)	
CKD group	666	23	4618.1	4.98	1.46 (0.90–2.37)	0.130	1.50 (0.92–2.45)	0.102

SSNHL, Sudden sensorineural hearing loss; CKD, Chronic kidney disease; CI, Confidence interval (*P < 0.05).

**Table 5:** Hazard ratios of sudden sensorineural hearing loss or Meniere’s disease by sex between patients with and without chronic kidney disease.

Variables	N	Case	Person-years	Incidence	Unadjusted HR (95% CI)	*P* value	Adjusted HR (95% CI)	*P* value
**SSNHL**								
**Sex Male**								
Comparison group	2682	20	28,926.4	0.69	1 (ref)		1 (ref)	
CKD group	1345	17	11,481.8	1.41	1.98 (1.04–3.79)*	0.038	1.99 (1.04–3.80)*	0.037
**Sex Female**								
Comparison group	2462	14	27,633.5	0.51	1 (ref)		1 (ref)	
CKD group	1227	14	10,874.0	1.29	2.30 (1.09–4.82)*	0.028	2.44 (1.16–5.14)*	0.019
**Meniere’s Diseases**								
**Sex Male**								
Comparison group	2682	81	28,823.3	2.81	1 (ref)		1 (ref)	
CKD group	1345	30	11,481.6	2.61	1.17 (0.76–1.79)	0.470	1.30 (0.85–1.99)	0.231
**Sex Female**								
Comparison group	2462	109	27,349.4	3.99	1 (ref)		1 (ref)	
CKD group	1227	51	10,799.0	4.72	1.47 (1.05–2.07)*	0.025	1.56 (1.11–2.20)*	0.010

SSNHL, Sudden sensorineural hearing loss; CKD, Chronic kidney disease; CI, Confidence interval (*P < 0.05).

## Discussion

CKD is a major global health burden due to its high prevalence, economic cost, and harmful effects on other organs. In this longitudinal study, we examined the association between CKD and SSNHL or Ménière’s disease in 2572 patients with CKD and 5144 sociodemographically matched comparison participants, whose data were extracted from a nationwide 8-year longitudinal cohort database of 1,025,340 South Korean patients. Interestingly, we observed that patients with CKD had a significantly increased incidence of SSNHL and Ménière’s disease, with an HR of 2.15 and 1.45, respectively, after adjusting for sociodemographic factors and the presence of comorbidities. Moreover, we observed that middle-aged patients with CKD had a significantly increased risk of SSNHL and Ménière’s disease. Further, the risk of developing SSNHL was higher in female patients with CKD than in male patients.

Research has shown that patients with CKD are prone to developing otologic symptoms related to audiovestibular dysfunction. These symptoms are often permanent, difficult to control, and have a significantly negative influence on the patient’s quality of life. Among the described audiovestibular dysfunctions, the possible cause linking CKD with SSNHL and Ménière’s disease remains unclear. However, the nephrons of the kidney and the stria vascularis of the cochlea show very similar anatomical, physiological, and pharmacological characteristics^17^. Additionally, antibodies formed against the nephrons may be immunologically deposited in the stria vascularis of the cochlea^18,19^. Moreover, CKD-related electrolytic and osmotic alterations that affect the cochlea can influence the labyrinth^13^. Furthermore, hemodialysis and renal transplantation may induce electrolyte disturbances and osmotic alterations in the inner ear, resulting in sensorineural hearing loss, tinnitus, and vertigo^9^. Prolonged hemodialysis may also result in the accumulation of amyloid in the inner ear tissues^20^. Thus, despite the exact pathogenesis of SSNHL and Ménière’s disease in patients with CKD remaining unclear, the suggested potential etiologic factors may explain the relationship between CKD and the subsequent development of SSNHL and Ménière’s disease.

To the best of our knowledge, this is the first study to investigate the association of developing SSNHL and Ménière’s disease in patients with CKD. Interestingly, we observed that CKD resulted in a significantly increased risk of SSNHL and Ménière’s disease. Consistent with our results, previous studies have shown that patients with CKD are at greater risk of tinnitus, SSNHL, and vestibular dysfunction than the normal population^15,16,21^. However, we also observed that middle-aged patients with CKD exhibited significantly increased risks of SSNHL and Ménière’s disease. Previous epidemiological studies have shown that SSNHL and Ménière’s disease have peak ages ranging between 30 and 60 years^22,23^. Thus, we consider that in middle-aged patients, the neuro-otologic organs may be more vulnerable to disturbances in water and electrolyte homeostasis. Additionally, we detected an increased risk of SSNHL in female patients with CKD compared to male patients. It remains unclear why female patients with CKD exhibited a significantly increased risk of SSNHL. However, another population-based study in South Korea showed that SSNHL has a slight female preponderance^24^; thus, we thought our finding may be due to this different sex preponderance.

This study has several unique strengths. First, we used a large national population-based database, which enabled us to effectively analyze all incidences of SSNHL and Ménière’s disease. Second, our cohort had a relatively long follow-up period (8 years). Third, the inclusion criteria of this study were based on an established diagnostic code, with the additional requirement of pure-tone audiometry for diagnosis. Finally, a prior study for validation of the KNHIS-NSC data revealed that the prevalence of 20 major diseases was similar for each year; thus, the reliability of the KNHIS-NSC data was defined as “fair to good.” Therefore, our findings suggest that CKD increases the risk of SSNHL and Ménière’s disease.

Our study also has some notable limitations. First, we could not obtain any specific personal health data, including body mass index, GFR level, lipid profiles, and information regarding behavioral risk factors, such as smoking or alcohol consumption. Second, SSNHL and Ménière’s disease were diagnosed based on the diagnostic code, which might be less accurate compared to the data obtained from medical charts that included details such as the medical history, imaging findings, or audiometry results. Third, we could not access the specific data such as the severity of hearing and vestibular impairment due to lacking in our registry; therefore, we were unable to investigate whether CKD influenced the severity of SSNHL and Ménière’s disease. Finally, family history, genetic conditions, and radiographic findings for SSNHL (such as enlarged vestibular aqueducts) could affect the potential for SSNHL and Ménière’s disease. However, in this cohort study, we could not include these variables as control variables, because our national insurance service does not cover these findings. Future clinical studies that investigate a wider range of factors and diagnostic criteria are needed to provide additional evidence for the link between CKD and SSNHL or Ménière’s disease.

In conclusion, this study investigated the possible link between CKD and the prospective development of SSNHL and Ménière’s disease. Interestingly, we observed that patients with CKD had a significantly higher risk of developing SSNHL and Ménière’s disease than non-CKD patients during an 8-year follow-up period. This finding suggests that CKD may be a risk factor for the development of SSNHL and Ménière’s disease; therefore, clinicians should consider patients with CKD to be at a high risk of developing SSNHL and Ménière’s disease and take specific measures to reduce the risk of developing these sequelae.

## Methods

### Data source and study population

This nationwide propensity score-matched cohort study was reviewed and approved by the Institutional Review Board of Hallym Medical University Chuncheon Sacred Hospital (Chuncheon, Korea). The Institutional Review Board of Hallym Medical University Chuncheon Sacred Hospital waived the written informed consent for this study due to the KNHIS-NSC dataset consists of de-identified secondary data for research purposes. This study adhered to the tenets of the Declaration of Helsinki. All citizens in Korea are obligated to enroll in the KNHIS, and a centralized large database provides access to nearly all data of the health insurance system. Therefore, the KNHIS contains reimbursement records from all medical facilities, including hospitals, private clinics, and public centers in South Korea. Claims are accompanied by data regarding diagnostic codes, procedures, prescription drugs, personal information about the patient, information about the hospital, the direct medical costs of both inpatient and outpatient care, and dental services. This study utilized the data of a representative sample of 1,025,340 adults from the 2002–2013 KNHIS-NSC in South Korea. This dataset accounted for approximately 2.2% of the South Korean population in 2002^25,26^. Stratified random sampling was performed using 1476 strata by age (18 groups), sex (2 groups), and income level (41 groups: 40 health insurance groups and 1 medical aid beneficiary) among the South Korean population. There were no duplicated or omitted patient health care records as all South Korean residents receive a unique identification number at birth.

### Study setting and participants

In this study, all disease diagnostic codes were identified using the International Classification of Diseases, 10th revision (ICD-10), Clinical Modification codes. The study design was a retrospective, nationwide propensity score-matched cohort study. The CKD group included all patients who received inpatient or outpatient care for an initial diagnosis of CKD (N18.1–5, N18.9) between January 2002 and December 2005. To further improve the accuracy of the CKD definition, we only included patients who had been diagnosed with CKD more than three times between 2002 and 2005. Patients were excluded if they (1) were diagnosed with SSNHL or Ménière’s disease between 2002 and 2005, (2) died as a result of any cause between 2002 and 2005 or as a result of an accident after 2006, and (3) were aged < 18 years. In the total 1,025,340 patient datasets, the number of CKD patients was identified using the ICD-10 code (N18.1–5, N18.9). Before selection, there were 11,382 CKD patients in the overall population (2002–2013). Among them, there were 3090 CKD patients in the period we decided to observe (2002–2005). A group of 2572 CKD patients was finally determined, excluding deaths and patients previously diagnosed with SSNHL and Ménière’s disease. The comparison group (non-CKD) comprised randomly selected propensity score-matched patients without CKD from the remaining cohort registered in the database (two for each patient with CKD). These patients were matched with patients with CKD for sociodemographic factors (age, sex, residential area, and household income), comorbidities, and the year of enrollment (CKD diagnosis). Eventually, 2572 and 5144 participants were enrolled in the CKD and comparison groups, respectively. All patients were monitored for the development of SSNHL or Ménière’s disease until December 2013. In this study, the endpoints of the study are the event (SSNHL or Ménière’s disease) or all-cause mortality. However, if patients had no events and were alive on December 31, 2013 (the final following period of this database) we censored this time point.

### Study outcome

The risk of SSNHL or Ménière’s disease development was the main study outcome. Incidence of SSNHL or Ménière’s disease during the follow-up was defined as the presence of ICD-10 codes H91.2 (SSNHL) or H81.0 (Ménière’s disease) with additional pure-tone audiometry more than twice.

### Independent variables

The study population was divided into two groups according to sex, three groups according to age (< 45, 45–64, and > 64 years), three groups according to household income (low: 0–30%, middle: 30–70%, and high: 70–100% of the median), and three groups according to area of residence (Seoul: the largest metropolitan region in South Korea, 2nd area: other metropolitan cities in South Korea, and 3rd area: small cities and rural areas). We also analyzed HTN and DM as comorbidities using the ICD-10 codes and prescription lists from the KNHIS-NSC database. HTN was defined as a previous diagnosis of hypertension (I10–11) and the use of antihypertensive drugs. DM was defined as a previous DM diagnosis (E10–14) and the use of one or more oral hypoglycemic agents or insulin. We defined the presence of comorbidities as any diagnoses of these codes between 2003 and 2004 prior to the diagnosis of SSNHL or Ménière’s disease.

### Statistical analysis

The risks of SSNHL and Ménière’s disease were compared between the CKD and comparison groups as person-years at risk, which were defined as the duration between either the date of CKD diagnosis or January 1, 2003 (for the comparison group), and the patient’s respective endpoint. Incidence rates per 1000 person-years for SSNHL or Ménière’s disease were obtained by dividing the number of patients with incidence of specific diseases by person-years at risk. Person-years consisted of the following 3 cases: (1) In the case of death, the number of years from the date of initial diagnosis of CKD to the date of death; (2) In the case of side effects, the number of years from the first diagnosis of CKD to the date of the first diagnosis of side effects; (3) If there are no side effects, the number of years from the date of initial diagnosis of CKD to ‘2013-12-31’, the study endpoint. To identify whether CKD increased the risk of occurrence of specific diseases, we used Cox proportional hazards regression analyses to calculate the HRs and 95% CIs, adjusted for other predictor variables. All statistical analyses were performed using R (version 3.5.0; R Foundation for Statistical Computing, Vienna, Austria), with a significance level of 0.05.

## References

[1] Webster AC, Nagler EV, Morton RL, Masson P (2017). Chronic kidney disease. Lancet.

[2] Hill NR (2016). Global prevalence of chronic kidney disease—A systematic review and meta-analysis. PLoS ONE.

[3] Go AS, Chertow GM, Fan D, McCulloch CE, Hsu CY (2004). Chronic kidney disease and the risks of death, cardiovascular events, and hospitalization. N. Engl. J. Med..

[4] Kundhal K, Lok CE (2005). Clinical epidemiology of cardiovascular disease in chronic kidney disease. Nephron. Clin. Pract..

[5] Foley RN (2010). Clinical epidemiology of cardiovascular disease in chronic kidney disease. J. Ren. Care.

[6] Etgen T, Chonchol M, Förstl H, Sander D (2012). Chronic kidney disease and cognitive impairment: A systematic review and meta-analysis. Am. J. Nephrol..

[7] Zammit AR, Katz MJ, Bitzer M, Lipton RB (2016). Cognitive impairment and dementia in older adults with chronic kidney disease: A review. Alzheimer Dis. Assoc. Disord..

[8] Perlman RL (2005). Quality of life in chronic kidney disease (CKD): A cross-sectional analysis in the Renal Research Institute-CKD study. Am. J. Kidney Dis..

[9] Krajewska WJ, Krajewski W, Zatoński T (2020). Otorhinolaryngological dysfunctions induced by chronic kidney disease in pre- and post-transplant stages. Eur. Arch. Otorhinolaryngol..

[10] Meena RS, Aseri Y, Singh BK, Verma PC (2012). Hearing loss in patients of chronic renal failure: A study of 100 cases. Indian J. Otolaryngol. Head Neck Surg..

[11] Jamaldeen J, Basheer A, Sarma AC, Kandasamy R (2015). Prevalence and patterns of hearing loss among chronic kidney disease patients undergoing haemodialysis. Australas. Med. J..

[12] Peyvandi A, Roozbahany NA (2013). Hearing loss in chronic renal failure patient undergoing hemodialysis. Indian J. Otolaryngol. Head Neck Surg..

[13] Gabr TA, Kotait MA, Okda HI (2019). Audiovestibular functions in chronic kidney disease in relation to haemodialysis. J. Laryngol. Otol..

[14] Mitschke H, Schmidt P, Zazgornik J, Kopsa H, Pils P (1977). Effect of renal transplantation on uremic deafness: A long-term study. Audiology.

[15] Kang SM, Lim HW, Yu H (2018). Idiopathic sudden sensorineural hearing loss in dialysis patients. Ren. Fail..

[16] Jung DJ, Lee KY, Do JY, Kang SH (2017). Chronic kidney disease as a risk factor for vestibular dysfunction. Postgrad. Med..

[17] Quick CA, Fish A, Brown C (1973). The relationship between cochlea and kidney. Laryngoscope.

[18] Gatland D, Tucker B, Chalstrey S, Keene M, Baker L (1991). Hearing loss in chronic renal failure-hearing threshold changes following haemodialysis. J. R. Soc. Med..

[19] Thodi C, Thodis E, Danielides V, Pasadakis P, Vargemezis V (2006). Hearing in renal failure. Nephrol. Dial. Transplant..

[20] Scarpioni R, Ricardi M, Albertazzi V, De Amicis S, Rastelli F, Zerbini L (2016). Dialysis-related amyloidosis: Challenges and solutions. Int. J. Nephrol. Renovasc. Dis..

[21] Shih CP (2017). Increased risk of tinnitus in patients with chronic kidney disease: A nationwide, population-based cohort study. PLoS ONE.

[22] Megighian D, Bolzan M, Barion U, Nicolai P (1986). Epidemiological considerations in sudden hearing loss: A study of 183 cases. Arch. Otorhinolaryngol..

[23] Bruderer SG, Bodmer D, Stohler NA, Jick SS, Meier CR (2017). Population-based study on the epidemiology of Ménière’s disease. Audiol Neurootol..

[24] Kim SH, Kim SJ, Im H, Kim TH, Song JJ, Chae SW (2017). A trend in sudden sensorineural hearing loss: Data from a population-based study. Audiol. Neurootol..

[25] Seong SC (2017). Cohort profile: the National Health Insurance Service-National Health Screening Cohort (NHIS-HEALS) in Korea. BMJ Open.

[26] Lee J, Lee JS, Park SH, Shin SA, Kim K (2017). Cohort profile: The National Health Insurance Service-National Sample Cohort (NHIS-NSC), South Korea. Int. J. Epidemiol..

